# Acute Adrenal Crisis Following Etomidate Administration in a Patient With Preexisting Adrenal Insufficiency

**DOI:** 10.7759/cureus.88003

**Published:** 2025-07-15

**Authors:** Radhika Mathur, Avetis Boyadzhyan, Annalee Mora, Shaikh Afaq

**Affiliations:** 1 Internal Medicine, HCA Florida Oak Hill Hospital, Brooksville, USA; 2 Anesthesiology, HCA Florida Oak Hill Hospital, Brooksville, USA

**Keywords:** adrenal crisis, adrenal insufficiency, etomidate, rapid sequence intubation, sepsis, stress-dose steroids

## Abstract

We present the case of a 79-year-old male with iatrogenic adrenal insufficiency on chronic hydrocortisone who presented with acute hypoxic respiratory failure due to severe bilateral pneumonia. He underwent rapid sequence intubation with IV etomidate and rocuronium. Within hours, he developed worsening hypotension, progressing to pulseless electrical activity cardiac arrest. Return of spontaneous circulation was achieved after two rounds of CPR and epinephrine. Despite vasopressor support, hypotension persisted until a 100 mg IV bolus of hydrocortisone was administered, leading to rapid hemodynamic stabilization. He was subsequently transitioned to stress-dose steroids. Given his underlying adrenal insufficiency, recent etomidate use, and sepsis, adrenal crisis was identified as the likely cause of his refractory shock and arrest. This case highlights the risk of etomidate-induced adrenal suppression in vulnerable patients and underscores the importance of administering corticosteroids prior to etomidate use, particularly in those with adrenal dysfunction or suspected sepsis.

## Introduction

Etomidate is commonly used for induction in critically ill patients due to its rapid onset, short duration, and hemodynamic stability [1]. However, it potently inhibits adrenal 11β-hydroxylase, a key enzyme in cortisol synthesis, leading to adrenal suppression even after a single dose [1]. While typically transient in healthy individuals, this effect can be clinically significant during physiological stress, especially in patients with adrenal insufficiency or sepsis [2]. In children with meningococcal sepsis, adrenal dysfunction has been shown to persist for at least 24 hours following a single dose [2]. Compared to alternatives like ketamine, etomidate maintains greater cardiovascular stability during rapid sequence intubation (RSI), which contributes to its frequent use [3]. Nonetheless, a meta-analysis of over 4,000 septic patients associated etomidate with increased adrenal insufficiency and a 20% higher relative risk of mortality [4]. Multiple studies have confirmed cortisol suppression after etomidate administration [5-9], and one randomized trial demonstrated that corticosteroid supplementation may help mitigate this effect [7]. The duration of suppression may extend beyond 24 hours, further underscoring the need for caution in high-risk patients [8,9]. A prospective study also found that even a single dose of etomidate during emergency intubation can affect both adrenal function and hemodynamics [10].

We present a case in which etomidate-induced adrenal suppression precipitated adrenal crisis in a patient with known adrenal insufficiency. This highlights the importance of careful agent selection and supports stress-dose corticosteroid administration in patients with sepsis or adrenal dysfunction.

## Case presentation

**Figure 1:**
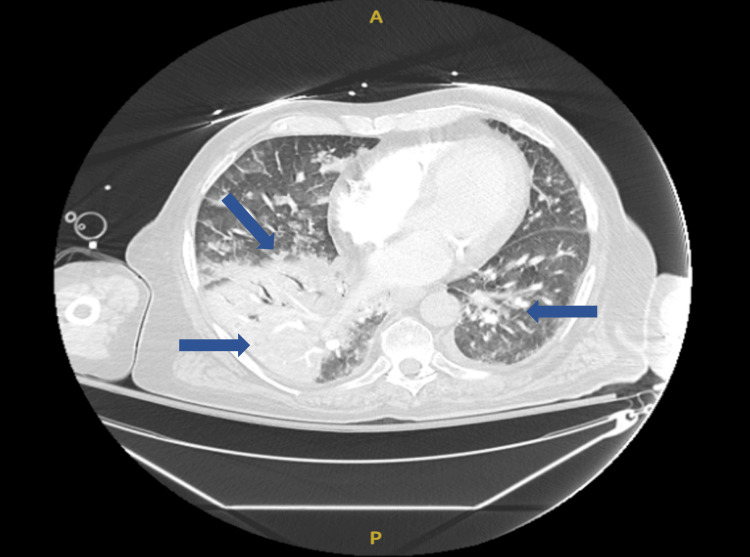
Dense consolidation of right lower lobe with air bronchograms consistent with pneumonia, patchy airspace opacities in bilateral upper lobes, right middle lobe, and left lower lobe representing pneumonia and/or pulmonary edema

A 79-year-old male with a past medical history of rheumatoid arthritis, psoriasis, hypertension, hypothyroidism, coronary artery disease status post percutaneous coronary intervention to the right coronary artery in 2020, benign prostatic hyperplasia, gastroesophageal reflux disease, generalized anxiety disorder, lumbar facet arthropathy with associated peripheral neuropathy, and iatrogenic adrenal insufficiency due to chronic steroid use for autoimmune conditions presented to the emergency department with acute hypoxic respiratory failure. CT angiography of the chest confirmed severe bilateral pneumonia (Figure 1).

At baseline, the patient was taking hydrocortisone 25 mg daily - administered as 20 mg in the morning (four 5 mg tablets) and 5 mg four hours later - along with methotrexate 2.5 mg daily, amlodipine 5 mg daily, levothyroxine 50 mcg daily, clopidogrel 75 mg daily, aspirin 81 mg daily, isosorbide mononitrate (Imdur) 30 mg daily, rosuvastatin (Crestor) 10 mg daily, alfuzosin 10 mg daily, pantoprazole 40 mg daily, alprazolam (Xanax) 0.5 mg as needed, hydrocodone-acetaminophen (Norco) 5-325 mg twice daily as needed, and gabapentin 300 mg three times daily.

**Figure 2:**
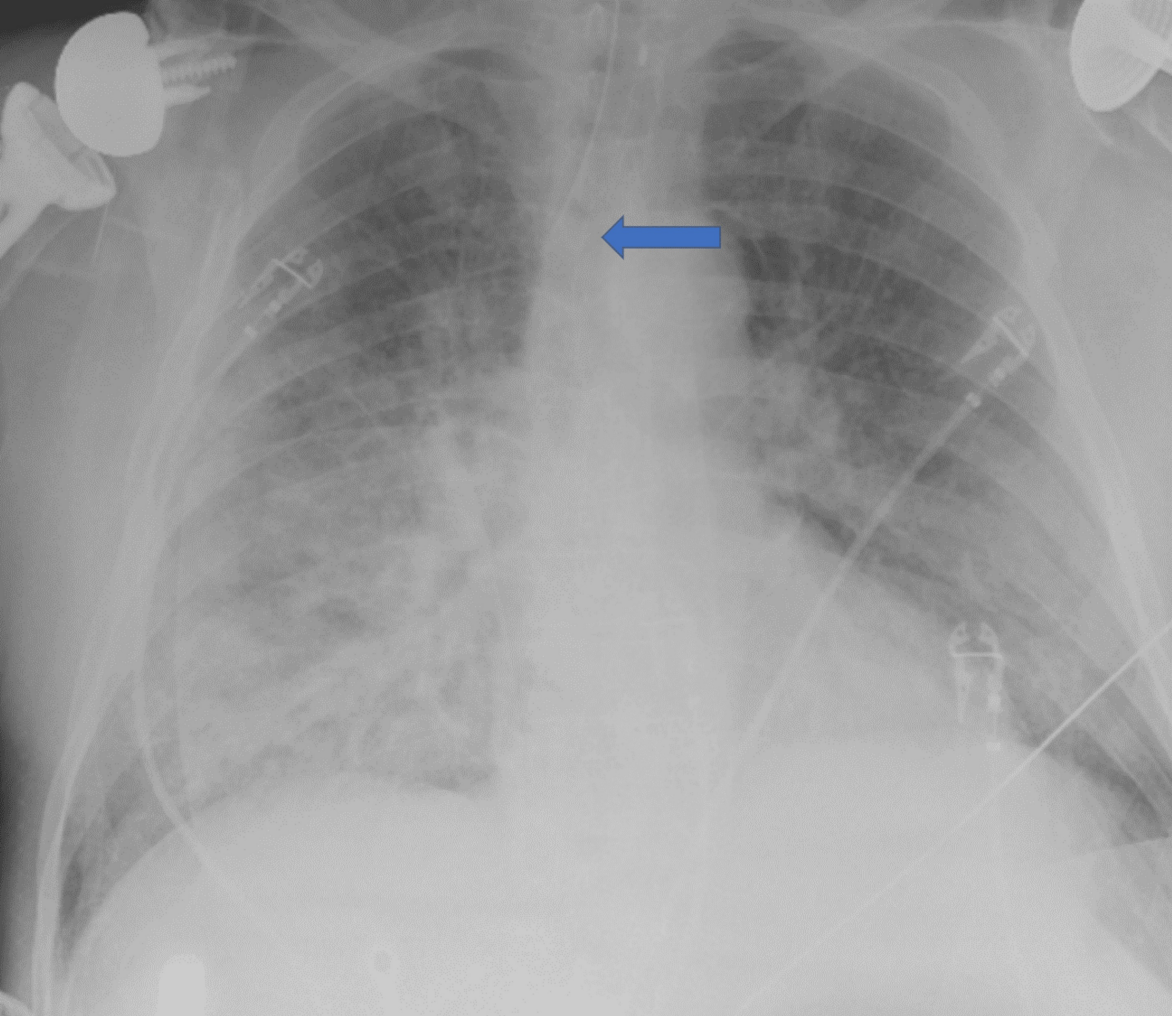
Post-intubation chest radiograph confirming endotracheal tube positioning

He was brought in by EMS for hypoxia, with a reported oxygen saturation of 86% in the field. Intubation was initially attempted but was unsuccessful; an iGel airway was placed as a temporizing measure. Upon arrival, he was in sinus rhythm with a heart rate of 97 bpm and blood pressure of 190/148 mmHg but remained critically hypoxic. There were no signs of rash, bronchospasm, or urticaria. Emergent RSI was performed using weight-based dosing of IV etomidate 20 mg (0.2 mg/kg) and rocuronium 50 mg (0.5 mg/kg) for his 96 kg body weight. Sedation was maintained with a titratable propofol infusion. A Macintosh 4 laryngoscope was used for intubation, and a 7.5 mm endotracheal tube was successfully placed at 23 cm at the lip. Endotracheal placement was confirmed by positive colorimetric change on end-tidal CO₂ monitoring, and post-intubation chest radiography demonstrated appropriate tube positioning (Figure 2). Post-intubation, his oxygen saturation improved to 99%, and arterial blood gas revealed a pH of 7.20, pCO₂ of 55.3 mmHg, and pO₂ of 141.3 mmHg on assist-control ventilation with 100% FiO₂ and a PEEP of 5 cm H₂O.

Despite sedation with propofol, the patient appeared visibly distressed, prompting initiation of a fentanyl infusion for analgesia. There was no evidence of patient-ventilator dyssynchrony or auto-PEEP. Shortly after starting the fentanyl drip, the patient appeared more comfortable. Initial laboratory results revealed a lactic acid level of 4.3 mmol/L, a WBC count of 11.7 × 10⁹/L (rising to 28.7 × 10⁹/L the following day), sodium 133 mmol/L, potassium 4.5 mmol/L, and glucose 146 mg/dL. A serum cortisol level was not obtained due to the patient’s known diagnosis of adrenal insufficiency. Troponin levels were elevated at 130 > 1,236 > 6,089 ng/L, prompting initiation of a heparin infusion and cardiology consultation. CT angiography of the chest demonstrated severe bilateral pulmonary consolidations concerning for pneumonia and possible pulmonary edema; pulmonary embolism was ruled out. Given the radiographic findings and clinical status, the patient met criteria for sepsis and was treated with 2 g IV cefepime and 1 g IV vancomycin. A 250 mL bolus of normal saline was administered, limited due to concern for pulmonary edema.

Approximately two hours post-intubation, the patient’s blood pressure decreased to 101/65 mmHg, while oxygen saturation remained stable at 97% on unchanged ventilator settings, with no evidence of auto-PEEP. Despite a dose reduction of sedatives, hypotension progressed to 57/39 mmHg approximately four hours later, culminating in a cardiac arrest. CPR was initiated per Advanced Cardiac Life Support protocol. Two cycles of chest compressions and epinephrine were administered, achieving return of spontaneous circulation after the second cycle. Persistent hypotension (BP 57/39 mmHg) necessitated initiation of a norepinephrine (Levophed) infusion.

Given the patient’s known adrenal insufficiency, recent administration of etomidate - a potent inhibitor of adrenal steroidogenesis - and concurrent sepsis, adrenal crisis was suspected as the likely cause of his refractory hypotension and cardiac arrest. A 100 mg IV bolus of hydrocortisone was administered as stress-dose steroid therapy, resulting in marked hemodynamic improvement and blood pressure stabilization with ongoing vasopressor support. He was subsequently transitioned to hydrocortisone 50 mg IV every six hours for five days, after which his outpatient regimen was resumed.

Later in his hospitalization, the patient developed atrial fibrillation with rapid ventricular response, managed with an amiodarone bolus followed by a continuous infusion. He reverted to normal sinus rhythm within 24 hours. Given his CHA₂DS₂-VASc score of 4, anticoagulation with heparin was continued. After extubation, he underwent cardiac catheterization, which showed nonobstructive coronary artery disease and a preserved left ventricular ejection fraction of 65%. He was ultimately discharged on his home medications, with the addition of apixaban (Eliquis) 5 mg orally twice daily for stroke prevention.

## Discussion

**Figure 3:**
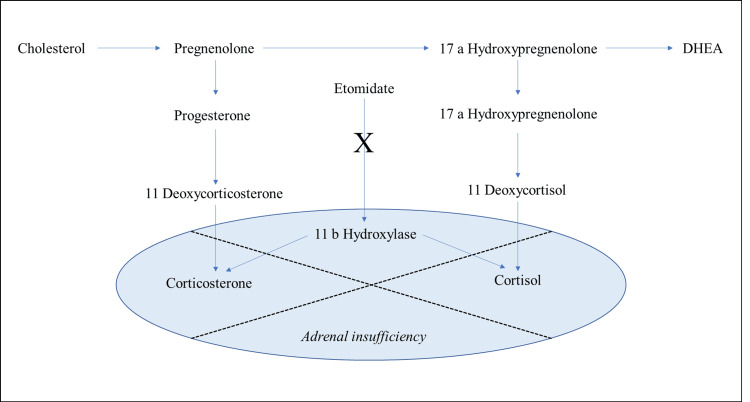
Mechanism of adrenal suppression following etomidate administration

Etomidate is often favored for emergent intubation due to its rapid onset and ability to preserve cardiovascular stability, making it especially useful in critically ill patients [1]. However, etomidate inhibits cytochrome P450 enzymes, particularly 11β-hydroxylase, leading to decreased cortisol synthesis and adrenal suppression (Figure 3) [1]. In healthy individuals, a single bolus typically causes only transient adrenal suppression without significant clinical effects. However, in patients with preexisting adrenal insufficiency or limited adrenal reserve, even one dose can precipitate adrenal crisis [2-4,10].

Sepsis further complicates this picture by increasing the physiological demand for cortisol and simultaneously impairing adrenal function [7]. This synergistic effect means that etomidate-induced adrenal suppression in septic patients can rapidly lead to refractory shock. Although randomized trials comparing etomidate to alternative agents like ketamine have not demonstrated mortality differences in general populations [3], these trials often exclude or underrepresent patients with adrenal insufficiency and sepsis. Meta-analyses focusing on septic patients have linked etomidate to increased rates of adrenal insufficiency and mortality [3,6].

In our patient, who had known adrenal insufficiency and severe sepsis due to bilateral pneumonia, etomidate likely compounded adrenal dysfunction, precipitating profound hypotension and cardiac arrest. Prompt recognition and treatment with stress-dose hydrocortisone were critical to reversing adrenal crisis and stabilizing hemodynamics. This case highlights the importance of considering both adrenal insufficiency and sepsis when selecting induction agents. When etomidate use is unavoidable in such high-risk patients, prophylactic corticosteroid supplementation should be administered to mitigate the risk of adrenal crisis [7].

## Conclusions

This case underscores that even a single bolus of etomidate can precipitate adrenal crisis in patients with preexisting adrenal insufficiency or additional risk factors, such as chronic glucocorticoid therapy for autoimmune diseases, critical illness with shock or sepsis, or any condition impairing hypothalamic-pituitary-adrenal axis function. Clinicians must carefully weigh the risks and benefits of etomidate in these vulnerable populations. When its use is unavoidable, alternative agents (e.g., ketamine) should be considered, or prophylactic stress-dose corticosteroids administered to reduce the risk of adrenal decompensation. Close monitoring is essential, with vigilance for clinical signs such as fatigue, weakness, nausea, vomiting, abdominal pain, and altered mental status, as well as laboratory findings including refractory hypotension, hypoglycemia, hyponatremia, and hyperkalemia. Prompt supportive care - including hemodynamic stabilization and corticosteroid replacement - is critical to preventing life-threatening complications.
